# *In vitro*-induction of metronidazole-resistant *Giardia duodenalis* is not associated with nucleotide alterations in the genes involved in pro-drug activation

**DOI:** 10.1590/0074-02760200303

**Published:** 2020-11-02

**Authors:** Luiz Antonio Pimentel Lopes-Oliveira, Maria Fantinatti, Alda Maria Da-Cruz

**Affiliations:** 1 Fundação Oswaldo Cruz-Fiocruz, Instituto Oswaldo Cruz, Laboratório Interdisciplinar de Pesquisas Médicas, Rio de Janeiro, RJ, Brasil; 2 Universidade do Estado do Rio de Janeiro, Faculdade de Ciências Médicas, Disciplina de Parasitologia, Rio de Janeiro, RJ, Brasil

**Keywords:** Giardia duodenalis, metronidazole, resistance

## Abstract

Giardiasis is an infectious disease caused by *Giardia duodenalis*. The pro-drug metronidazole (MTZ) is the first-line treatment for giardiasis. Parasite’s proteins as pyruvate:ferredoxin oxidoreductase (PFOR), ferredoxin (Fd), nitroreductase-1 (NR-1) and thioredoxin reductase (TrxR) participate in MTZ activation. Here, we showed *Giardia* trophozoites long-term exposed to MTZ presented higher IC_50_ than controls, showing the drug influenced the parasite survival. That reduction in MTZ’s susceptibility does not seem to be related to mutations in the genes *pfor*, *fd*, *nr-1* or *trxr*. It points that different mechanism as alterations in other metabolic pathways can account for *Giardia* resistance to MTZ therapy.

Giardiasis is a waterborne disease caused by the protozoan *Giardia duodenalis* (syn. *G. lamblia*, *G. intestinalis*) that colonises the small intestine. The infection is worldwide distributed affecting around 280 million people each year most of them children.^1^ ^,^ ^2^ ^)^ Although the prevalence of giardiasis is high in low income regions, the transmission is also a concern in developed countries, especially as outbreaks.^2^ ^)^ *G. duodenalis* is a zoonotic parasite classified into eight assemblages (A to H).^3^ ^)^ The assemblages A and B are classically known to infect humans and dogs. In addition, the assemblage E infecting humans was also described in Brazil^4^ ^)^ and Australia.^5^ ^)^ The faecal-oral transmission along with the close interaction with pets increase the risk of *G. duodenalis* infection

There are relatively few therapies available for treatment of giardiasis and 5-nitroimidazoles drugs, mainly metronidazole (MTZ), are first-line treatment.^6^ ^,^ ^7^ MTZ is a pro-drug and need to be metabolised to exert biological activity. In *G. duodenalis*, the proteins pyruvate:ferredoxin oxidoreductase (PFOR) and ferredoxin (Fd) act together to activate MTZ.^8^ ^)^ However, two other proteins were also associated with MTZ activation: nitroreductase-1 (NR-1)^9^ ^)^ and thioredoxin reductase (TrxR).^10^ ^)^ MTZ activation generates a nitro anion radical that causes oxidative damage to several cellular macromolecules, especially DNA and proteins, leading to the parasite’s death.^7^ ^,^ ^11^

The use of 5-nitroimidazoles is well established in clinical practice, the efficacy is high and the estimate cure rate can achieve up to 90% of the giardiasis treated cases.^6^ However, increasing therapeutic failure has been observed in clinical practice. Studies conducted in Spain^12^ ^,^ ^13^ and United Kingdom^14^ evaluating travellers point to increasing rates of 5-nitroimidazole refractory *Giardia* infection. Moreover, in a Cuban cohort of 456 *Giardia*-microscopically positive individuals, 46% of them experienced therapeutic failure after the first course of MTZ treatment.^15^ ^)^ Indiscriminate use of antiparasitic drugs together with repeated treatments can increase the chances for rising MTZ-resistant *G. duodenalis* strains. These strains can spread into the environment, infect susceptible individuals and, consequently, make the treatment of giardiasis more difficult.

The resistance of microorganisms to different classes of drugs is a worldwide concern. Several mechanisms cause resistance to drugs, such as mutation, changes in gene expression and protein abundance, efflux pumps, epigenetic changes and others. *Giardia* resistance to 5-nitroimidazoles was extensively documented *in vitro*, but the mechanisms underlying this resistance is still largely unknown.^16^ ^-^ ^19^ Considering the role of PFOR, Fd, NR-1 and TrxR in the MTZ activation, nucleotide alterations in genes that encode these proteins could be associated with 5-nitroimidazoles parasitic resistance. Herein, we evaluated whether *in vitro* induced MTZ-resistant *G. duodenalis* strain presents mutations in these genes.

Trophozoites of *G. duodenalis* WB strain [ATCC50803] were grown in TYI-S-33 medium as previously described by Keister,^20^ supplemented with 10% of foetal bovine serum (Sigma Chemical Co., Saint Louis, USA) and incubated at 37°C.

Four groups were defined where *G. duodenalis* was continuously exposed to different concentrations of MTZ as follow: 5 µM (MTZ5); 10 µM (MTZ10); 20 µM (MTZ20); and 80 µM (MTZ80). Two groups were used as controls: the group no exposed to MTZ (SMTZ) and the group exposed to 0.05% (V/V) of dimethylsulfoxide (Sigma, USA) (CDMSO), the vehicle in which MTZ was solubilised. After 16 weeks, the IC_50_ was evaluated for all groups evaluated.^21^ Briefly, 5 × 10^4^ parasites from each experimental group were plated in a 96-well flat-bottom plate, in which a serial dilution of MTZ was carried out. The plates were incubated at 37°C for 72 h. Resazurin was added to evaluate the parasite’s viability. After 4 h incubation, the reaction was quantified by fluorimeter (λex 560 nm - λem 590 nm).^21^ The tests were performed in triplicate and the IC_50_ results were analysed using the GrafPad Prism 6.0 software (www.grafpad.com/scientific-software/prism/). The results were expressed as IC_50_ values and resistance factor, i.e, IC_50_ fold change in relation to control. The ANOVA test was used for comparative analysis of the IC_50_ of groups.

The DNA from experimental and control groups were extracted using DNAzol^TM^ reagent (Qiagen, Hilden, Germany) according to the manufacturer’s instructions. The DNA was subjected to PCR to amplify gene fragments. The specific primers for *fd* (orf. GL50803_9662), *nr-1* (orf.GL50803_6175) and *trxr* (orf.GL50803_9827) were designed using the NCBI Pick Primers Tool (https://www.ncbi.nlm.nih.gov/tools/primer-blast/). The amplified fragments of *nr-1* (953 base pairs), *fd* (251 base pairs) and *tr* (1,273 base pairs) comprised the complete sequence of these proteins. The amplified fragment of *pfor* (342 base pairs) (orf.GL50803_114608) contains the coding region of the catalytic site of the enzyme.^16^ ^)^ The primers sequences, the concentration of the mix reagents and amplification conditions are described in Table. The effectiveness and efficiency of PCR conditions were assessed by electrophoresis in agarose gel 1%.

**TABLE:** Oligonucleotide sequence of primers and amplification conditions of the polymerase chain reaction

Locus	Sequence(5’-3’)	MgCl_2_ (mM)	Taq (U)	*Primer* (µM)	Denaturation	Hybridisation	Extension	Cycles	Product size (bp)
*pfor*	fwd-TGCGGTTTCTGCTCTGTCCAG rev-GTTGCAGCTCTCCGTGTCGAT	2	1	1	94°C/5 min	70°C/30 s	72°C/5 min	35	342
*fd*	fwd- GACTTCCACCGCATTCGTA rev-AGAGAAGGCAGGCGTAGAGA	1.5	3	0.1	95°C/5 min	60°C/30 s	72°C/7 min	35	251
*nr-1*	fwd- AGCGCTGGTCTCTGTTTACC rev-AGTCAACACTTTTCTTCCGGTC	3	3	0.1	95°C/7 min	60°C/30 s	72°C/7 min	35	953
trxr	fwd-CTATAGCCCCGGACGCATTT rev-GTTTCAACATCCCCTCCCCC	2	3	0.1	95°C/7 min	62°C/30 s	70°C/7 min	35	1,273

bp: base pairs.

The gene fragments obtained in the PCR reaction were purified using the QIAquick^®^ - PCR purification Kit (Qiagen, Germany), according to the manufacturer’s instructions. The effectiveness and efficiency of PCR conditions were assessed by electrophoresis in agarose gel 1%. The purified fragments were subjected to sequencing using the BigDye^®^ Terminator v3.1 Cycle Sequencing Kit (Applied Biosystems, Foster City, USA). The precipitation and electrophoresis steps were performed at Sequencing Service DNA Sequencing Platform - Fundação Oswaldo Cruz.^22^

The electropherograms were analysed and their quality was verified by the Chromas 2.4 program (http://tecnelysium.com.au/wp/chormas/). The identity percentage was verified by the Basic Local Alignment Search Tool (BLASTn) (https: /blast.ncbi.nlm.nih.gov./Blast.cgi). The obtained sequences were aligned with the nucleotide sequences of *pfor*, *fd*, *nr-1* and *trxr* of *G. duodenalis* in GenBank using the CLUSTAL W algorithm^23^ in the Mega 7.0 package (http://www.megasoftware.net)^24^ and the consensus obtained by CAP3 Sequence Assembly Program (http://doua.prabi.fb/software/cap3).

To evaluate whether the MTZ exposition decreases the parasite’s susceptibility to the drug, *G. duodenalis* trophozoites were continuously exposed to different concentrations of the MTZ during 16 weeks. Afterwards, the parasite’s susceptibility to the different MTZ concentrations was evaluated by the rate of trophozoite replication determined by the IC_50_ values and the resistance factor (Figure). The IC_50_ values of MTZ5, MTZ10 and MTZ20 groups were significantly higher in comparison to SMTZ and CDMSO groups. This result indicates that *G. duodenalis* exposition to MTZ induced a reduction of the parasite’s susceptibility in a dose dependent manner. The IC_50_ values of MTZ80 group could not be determined since the majority of parasites were dead at 16 weeks*.*

**Figure:**
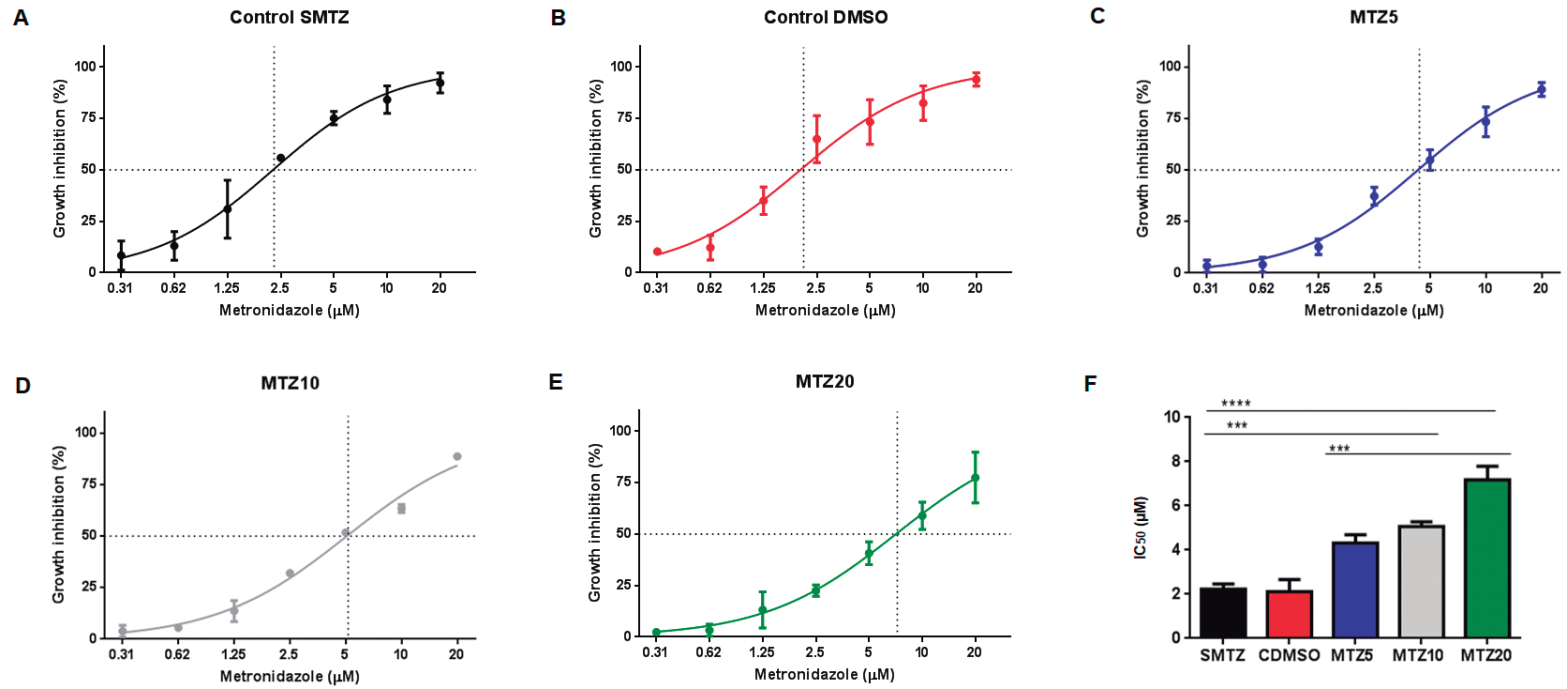
Evaluation of the susceptibility of *Giardia duodenalis* exposed *in vitro* to different concentrations of metronidazole (MTZ). The IC_50_ values are indicated on the curves by intersecting MTZ concentration (x-axis) with the 50% growth inhibition value (y-axis). The graphs are representative of three independent experiments and the results were expressed as mean and standard deviation. (A) No exposed to MTZ (SMTZ) (IC_50_ = 2.26 ± 0.3 µM); (B) exposed to dimethylsulfoxide (CDMSO) (IC_50_ = 2.02 ± 0.3 µM); (C) MTZ5 (IC_50_ = 4.3 ± 0.6 µM); (D) MTZ10 (IC_50_ = 5.07 ± 0.7 µM); (E) MTZ20 (IC_50_ = 7.09 ± 0.8 µM); (F) IC_50_ values of the groups are compared (ANOVA test). The resistant factor for MTZ5, MTZ10 and MTZ 20 cultures were, respectively, 2.1, 2.5 and 3.5. ***: p < 0.001; ****: p < 0.0001.

The reduced susceptibility of *G. duodenalis* trophozoites to MTZ could be caused by alterations in the nucleotide sequence of the genes associated with MTZ activation. Thus, nucleotide sequences of these genes were evaluated. All groups had their DNA amplified and sequenced. The length of the amplified fragments obtained by PCR are the expected for each amplified sequence. No alteration on the nucleotide sequence of *pfor*, *fd*, *nr-1* and *trxr* was identified in the parasites from the exposed groups MTZ5, MTZ10, MTZ20, MTZ80 and controls. These results indicate that the reduction of MTZ susceptibility of *Giardia* trophozoites *in vitro* is not a consequence of *pfor*, *fd*, *nr-1* and *trxr* nucleotide modifications.

Several factors can account for refractory cases of giardiasis after treatment with different drug classes, as inadequate use of drug doses, immunosuppression, reinfection and resistance of the parasite to drugs. In our study, we induced *G. duodenalis-*resistant trophozoites by *in vitro* exposition to different concentrations of MTZ and demonstrated that no nucleotide alteration occurred in genes previously associated with *Giardia* resistance.

The *in vitro* exposure of *G. duodenalis* to MTZ significantly increased the IC_50_ values of the groups exposed to this drug compared to controls, similar to the findings of other studies.^16^ ^,^ ^17^ ^,^ ^25^ Herein, the IC_50_ fold change values obtained from MTZ exposed parasites ranged from 2.01 to 3.51, as observed to other MTZ-resistant trophozoites strains.^17^ ^)^ However, our strategy of continuous MTZ exposition differed from other authors who used intermittent exposure of *G. duodenalis* to high concentrations of 5-nitroimidazoles or ultraviolet radiation followed by exposure to this drug.^25^ ^)^ As expected, the results demonstrated that the continuous exposure of *G. duodenalis* to MTZ decreased the parasite’s susceptibility to this drug *in vitro*.

The mechanism responsible for *G. duodenalis in vitro* resistance to MTZ is still unknown. Since parasite’s proteins such as PFOR, Fd, NR-1 and TrxR participate in the activation of MTZ, nucleotide changes in the genes that encode these proteins could prejudice the activation of the drug, leading to a reduction in *Giardia* susceptibility. This prompted us to evaluate the gene sequences of these proteins in strains of *G. duodenalis* resistant to MTZ *in vitro*. Unexpected, we did not find any nucleotide change in these analysed genes.

Analysis of transcriptome and proteome of resistant strains of *G. duodenalis* have shown great variability in gene expression and protein abundance of Fd and PFOR.^17^ ^,^ ^19^ However, some studies have detected a decrease in the PFOR enzyme activity^10^ ^,^ ^26^ or the Fd activity in MTZ-resistant *G. duodenalis* strains.^27^ ^)^ We extended previous studies that showed MTZ-resistant strains of *G. duodenalis* did not present any nucleotide changes in the gene region that encode PFOR catalytic site.^16^ Furthermore, the expression of *pfor* mRNA was shown to be higher in the resistant strain of *G. duodenalis* compared to controls.^16^ ^)^ These findings evidence the complexity involving MTZ-*G. duodenalis* resistance process, which may include another mechanisms. Then, nucleotide changes only in PFOR and Fd are insufficient to elucidate the resistance of *G.* *duodenalis* to MTZ.

Like Fd and PFOR, gene expression and protein abundance of TrxR showed variable results when MTZ-resistant *G. duodenalis* strains were evaluated.^17^ ^-^ ^19^ However, NR-1 was downregulated in three *G.* *duodenalis* strains resistant to MTZ,^17^ ^,^ ^19^ with evidence of *nonsense* mutation in NR-1 transcripts in one of strains.^17^ ^)^ This fact demonstrates the importance of evaluating the nucleotide sequence of this gene in different *in vitro* MTZ-resistant *G. duodenalis* strains. 

Indiscriminate use of antiparasitic drugs, a common practice in health care units, can select resistant strains of several microorganisms. Given the increase in therapeutic failure cases in the treatment of giardiasis with 5-nitroimidazoles, efforts must be made to elucidate which mechanisms may be responsible for the resistance of *G. duodenalis* to MTZ. In the present study, we demonstrated that the exposure of *G. duodenalis* to MTZ decreases the parasite’s susceptibility to the drug; however, this decrease could not be attributed to nucleotide changes in the *G. duodenalis* genes linked to MTZ activation. Our results points that different mechanisms as alterations in other metabolic pathways can account for *Giardia* resistance to MTZ therapy.
